# Mixed Active and Passive, Heart Rate-Controlled Heat Acclimation Is Effective for Paralympic and Able-Bodied Triathletes

**DOI:** 10.3389/fphys.2019.01214

**Published:** 2019-09-20

**Authors:** Ben T. Stephenson, Keith Tolfrey, Victoria L. Goosey-Tolfrey

**Affiliations:** ^1^The Peter Harrison Centre for Disability Sport, School of Sport, Exercise and Health Sciences, Loughborough University, Loughborough, United Kingdom; ^2^Physiology, English Institute of Sport, Loughborough Performance Centre, Loughborough University, Loughborough, United Kingdom

**Keywords:** disability, thermoregulation, isothermic, acclimatization, triathlon, elite

## Abstract

**Purpose:** The aims of this study are to explore the effectiveness of mixed active and passive heat acclimation (HA), controlling the relative intensity of exercise by heart rate (HR) in paratriathletes (PARA), and to determine the adaptation differences to able-bodied (AB) triathletes.

**Methods:** Seven elite paratriathletes and 13 AB triathletes undertook an 8-day HA intervention consisting of five HR-controlled sessions and three passive heat exposures (35°C, 63% relative humidity). On the first and last days of HA, heat stress tests were conducted, whereby thermoregulatory changes were recorded during at a fixed, submaximal workload. The AB group undertook 20 km cycling time trials pre- and post-HA with performance compared to an AB, non-acclimated control group.

**Results:** During the heat stress test, HA lowered core temperature (PARA: 0.27 ± 0.32°C; AB: 0.28 ± 0.34°C), blood lactate concentration (PARA: 0.23 ± 0.15 mmol l^−1^; AB: 0.38 ± 0.31 mmol l^−1^) with concomitant plasma volume expansion (PARA: 12.7 ± 10.6%; AB: 6.2 ± 7.7%; *p* ≤ 0.047). In the AB group, a lower skin temperature (0.19 ± 0.44°C) and HR (5 ± 6 bpm) with a greater sweat rate (0.17 ± 0.25 L h^−1^) were evident post-HA (*p* ≤ 0.045), but this was not present for the PARA group (*p* ≥ 0.177). The AB group improved their performance by an extent greater than the smallest worthwhile change based on the normal variation present with no HA (4.5 vs. 3.7%).

**Conclusions:** Paratriathletes are capable of displaying partial HA, albeit not to same extent as AB triathletes. The HA protocol was effective at stimulating thermoregulatory adaptations with performance changes noted in AB triathletes.

## Introduction

Competitive sporting events are commonly held in hot and/or humid environments; therefore, strategies are commonly sought to attenuate the deterioration typical of endurance performance in such conditions (Daanen et al., 2018); one such strategy that is commonly used by athletes is heat acclimation (HA). Heat acclimation can invoke myriad positive adaptations, which improve heat tolerance, including lower: core (*T*_c_) and skin (*T*_sk_) temperature; submaximal heart rate (HR); carbohydrate metabolism; and sweat electrolyte content. Additionally, it can lead to an increased sweat rate, plasma volume (PV) expansion, and positive perceptual alterations with a resultant improved performance in the heat (Corbett et al., 2014).

Heat acclimation typically involves daily or alternate days of heat stress over a 5–16-day period, whereby *T*_c_, *T*_sk_, and sweat rate are elevated for 1–2 h (Daanen et al., 2018). To provide a constant heat stress across HA, isothermic protocols have been employed, whereby the external workload is manipulated within- and between-HA sessions to maintain a *T*_c_ of ~38.5°C (Gibson et al., 2015; Neal et al., 2016; Ruddock et al., 2016). However, these approaches bring the financial burden of measuring *T*_c_ *via* ingestible sensors or participant discomfort from rectal temperature assessment. Furthermore, rectal temperature measurement poses a risk of autonomic dysreflexia in athletes with a spinal cord injury (Price and Campbell, 1999). Consequently, controlling HA intensity using HR has been proposed by Périard et al. (2015) as a practical method of maintaining a constant cardiovascular stimulus. Based on evidence that HR is unchanged through isothermic HA (Magalhães et al., 2010; Garrett et al., 2012; Zurawlew et al., 2015; Pethick et al., 2019), this method of controlling the relative intensity would result in a constant thermal load during HA. Initial evidence suggests that this approach may be efficacious in invoking HA in soccer players (Philp et al., 2017).

Despite the efficacy of HA for invoking thermoregulatory adaptations, commonly studied protocols may not be appropriate for elite athletes in preparation for competition, especially in a multi-modal sport such as triathlon. This is due to the protocols typically involving multiple days of exercise in the heat, which does not fit with the weekly training distribution of athletes tapering into competition (Mujika, 2011). As such, passive HA has recently been explored (Stanley et al., 2015; Zurawlew et al., 2015, 2018). Using post-exercise heat exposures, positive adaptations are achievable without excessive physical stress (Stanley et al., 2015; Zurawlew et al., 2015, 2018). Yet, while passive HA can stimulate positive responses, it has been questioned whether it can provide full heat adaptation (Périard et al., 2015; Tyler et al., 2016). As such, Guy et al. (2014) state that for athletes to optimally adapt to the heat, and in a time-efficient manner, protocols may best utilize a combination of active and passive HA.

While HA has been studied in a range of able-bodied (AB) athletes (Lorenzo et al., 2010; Garrett et al., 2012; Stanley et al., 2015; Ruddock et al., 2016), little attention has been paid to Paralympic athletes. These athletes are likely to be at heightened risk for performance decrements in the heat as a consequence of varied impairments in autonomic or behavioral thermoregulatory function (Webborn and Van de Vliet, 2012). In the sole published study of Paralympic athletes and HA, Castle et al. (2013) researched the adaptive potential of target shooters with a spinal cord injury. The athletes performed a 7-day protocol consisting of 20 min moderate intensity arm cranking followed by 40 min passive heat exposure and displayed several thermoregulatory adaptations. While this provided the first evidence of Paralympic athletes’ capability to adapt to the heat, even to a relatively modest heat stimulus, it is not known whether HA is also effective in endurance-trained Paralympic athletes. Further, it is not known how adaptations to HA in Paralympic athletes differ from AB individuals.

The aims of this study were to investigate the efficacy of a mixed active and passive HA protocol in the sport of paratriathlon. To negate the issue of potential cost and discomfort associated with isothermic protocols, a controlled relative intensity design was utilized by regulating exercise intensity using HR, which may be more applicable for elite athletes. A further aim was to determine how HA adaptations may differ between Paralympic and AB athletes.

## Materials and Methods

### Participants

Twenty-nine (22 males and 7 females) paratriathletes and triathletes were recruited to partake in the present study. From this pool, three separate groups were formed: a group of elite paratriathletes [PARA; *n* = 7; amputation *n* = 3, incomplete spinal cord injury *n* = 1 (wheelchair user), hemiplegia cerebral palsy *n* = 1, lower leg impairment *n* = 1, visual impairment *n* = 1]; an AB HA group (AB-ACC; *n* = 13); an AB control group (AB-CON; *n* = 9; Table 1). Participants trained at least five times per week. All provided written informed consent and the procedures were approved by the Loughborough University Ethical Advisory Committee (R16-P010). No participants reported being heat acclimated/acclimatized prior to the start of the study.

**Table 1:** Participant characteristics for the paratriathlon (PARA), able-bodied acclimation (AB-ACC), and able-bodied control (AB-CON) groups.

Parameter	PARA (four males, three females)	AB-ACC (nine males, four females)	AB-CON (eight males)
Age (y)	31 ± 9^*^	25 ± 7	21 ± 2
Body mass (kg)	67.8 ± 9.0	69.3 ± 9.4	70.0 ± 6.9
Cycling $\overset{Ë™}{V}O_{2\mathrm{peak}}$ (ml·kg^−1^·min^−1^)	57.7 ± 7.6	61.5 ± 6.4	62.7 ± 8.1
MAP (W)	324 ± 73	340 ± 74	379 ± 45
AeLT (W)	181 ± 48	187 ± 42	192 ± 28

$\overset{Ë™}{V}O_{2\mathrm{peak}}$, *peak rate of oxygen uptake; MAP, maximal aerobic power output; AeLT, aerobic lactate threshold power output*.

* *Significantly greater than AB-CON (*p* = 0.039)*.

### Study Design

**Figure 1:**
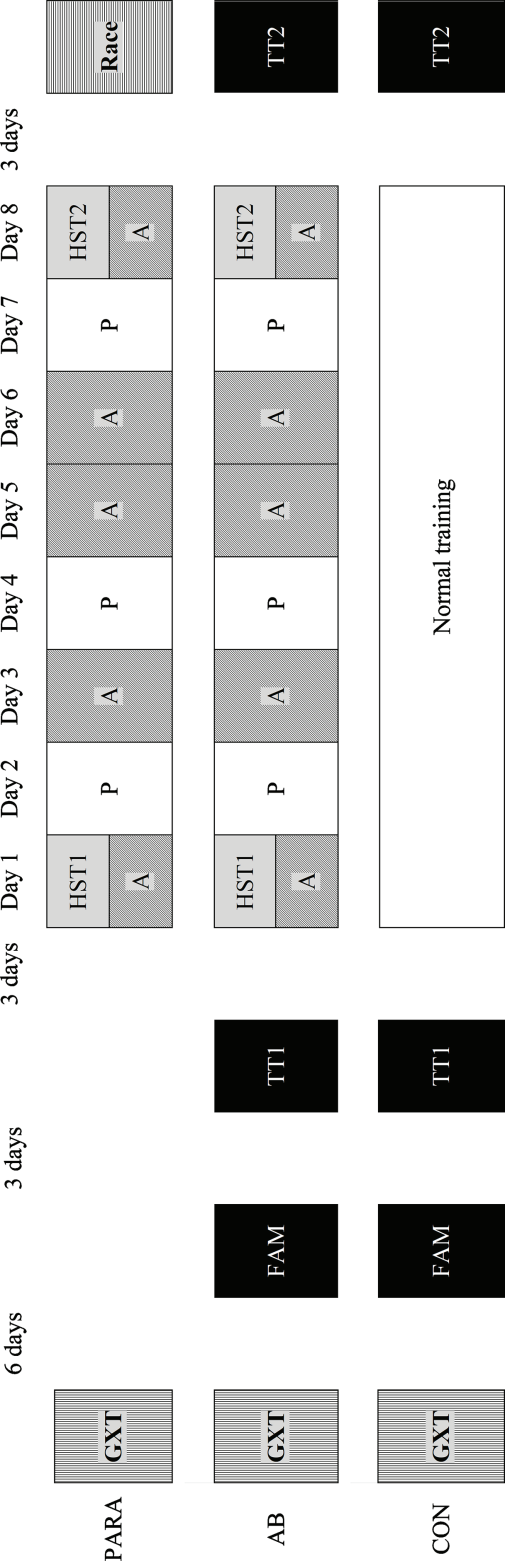
Schematic of the study protocol. PARA, paratriathlon group; AB-ACC, able-bodied acclimation group; AB-CON, able-bodied control group; GXT, graded exercise test; FAM, familiarization; TT, performance trial; HST, heat stress test; A, active heat acclimation; P, passive acclimation.

Both the PARA and AB-ACC groups undertook an 8-day HA period. Due to the nature of the paratriathletes’ pre-competition routine, it was not possible to gain a direct performance measure in this group. However, this was undertaken in the AB-ACC group pre- and post-HA. The AB-CON group solely undertook the performance trials with no HA to determine natural variation in performance (Figure 1).

During the study period, all participants were instructed to maintain their usual training routine. All testing was performed in the same geographical location with an average outdoor environmental temperature of 13.6 ± 6.2°C during the study period. Participants were free to drink *ad libitum* during all visits, but fluid intake was restricted to water. Participants were instructed to abstain from alcohol for 48 h before every trial while standardizing food, fluid, sodium, and caffeine intake. During every trial in the heat, participants were instructed to keep clothing consistent. All trials in the heat were conducted in an environmental chamber (Weiss Gallenkamp, Loughborough, United Kingdom; 35.1 ± 0.4°C, 63.4 ± 4.1% relative humidity) with a fan producing an airflow of 2.0 m s^−1^ at the body (5,400 FW, Kestrel Meters, Minneapolis, MN, United States). All trials were scheduled at the same time of day to limit the cofounding effect of circadian rhythm variation (Winget et al., 1985). Prior to all trials, participants first provided a urine sample for the determination of urine specific gravity (USG) by refractometer (PCE-032, PCE Instruments UK Ltd., Southampton, United Kingdom) before nude body mass was recorded *via* electronic scales (Adam Equipment Co. Ltd., Milton Keynes, United Kingdom). Participants with a USG ≥ 1.020 were advised to increase fluid intake pre-trial *via* ingesting 250–500 ml of water 30 min pre-trial. After the trials, nude body mass was again recorded after towel drying. Fluid intake was calculated from drinks bottle mass changes, and sweat loss was calculated from fluid intake and body mass changes. Sweat gain was calculated from sweat loss and *T*_c_, where available.

### Graded Exercise Tests

For all participants, the first trial consisted of a submaximal cycling graded exercise test for the determination of individuals’ aerobic lactate threshold (AeLT) with a maximal graded exercise test for the determination of maximum HR, maximum aerobic power output (PO), and peak rate of oxygen uptake. This was the only visit conducted in temperate ambient conditions. During both tests, participants cycled on the Cyclus 2 ergometer (RBM elektronik-automation GmbH, Leipzig, Germany), using their own bicycle, at incremental POs. AeLT was determined from athletes’ blood lactate concentration (BLa; Biosen C-Line, EKF Diagnostics, Magdeburg, Germany) and oxygen uptake (Metalyzer® 3B, Cortex Biophysik GmbH, Leipzig, Germany) using the methods of Beaver et al. (1985). HR was recorded continuously (Polar RS400, Polar, Kempele, Finland). Maximum HR, maximum aerobic PO, and peak rate of oxygen uptake were defined as the highest value recorded during any 5, 60, or 30 s epoch of the maximal test, respectively.

### Familiarization and Performance Trials

The performance trial consisted of a simulated 20 km cycling time trial in the heat. Six days after the graded exercise tests, participants were familiarized to the performance trial. The familiarization, pre-acclimation performance trial (TT1), and post-acclimation performance trial (TT2) were all performed on the Cyclus 2 ergometer. Warm-up for all performance trials was standardized to 10 min cycling at AeLT, followed by 5 min passive recovery. Participants were instructed to perform the test in the shortest amount of time possible with no encouragement given. During all trials, participants were blinded to all measures except distance covered. HR was recorded throughout, while capillary BLa was assessed pre-trial and every 5 km. Similarly, thermal sensation (TS) (Hardy, 1970) and rating of perceived exertion (RPE) (Borg, 1998) were collected every 5 km.

### Heat Stress Tests

Participants were first instructed to rest in a seated position of 10 min for the provision of fingertip capillary blood samples, as performed elsewhere (Castle et al., 2013; Stanley et al., 2015; Ruddock et al., 2016). In duplicate, samples were collected in hematocrit tubes (Hawksley, Sussex, United Kingdom) for the determination of hematocrit, while 20 μl samples were collected in capillary tubes (EKF Diagnostics) for the measurement of hemoglobin. After blood samples were collected, participants were fitted with temperature loggers (DS1922L Thermochron iButton^®^, Maxim Integrated Products, Inc., Sunnyvale, CA, United States) using surgical tape to measure *T*_sk_ at four sites (*pectoralis major* muscle belly, lateral head of *triceps brachii*, *rectus femoris* muscle belly, and lateral head of the *gastrocnemius*) (Ramanathan, 1964), on the right side of the body, at a recording rate of 30 s. Subsequently, sweat patches (Tegaderm +Pad, 3 M, St. Paul, MN, United States) were placed at four sites (forearm, chest, upper back, and thigh), after cleaning with deionized water, for the collection of localized sweat on the left side of the body. The heat stress test (HST) consisted of 10 min standardized fixed intensity cycling at an intensity equating to 80% AeLT before immediately starting 40 min fixed intensity cycling at participants’ AeLT. During the HST, *T*_c_ was recorded at 5-min intervals *via* ingestible *T*_c_ sensor (CorTemp, HQ Inc., Palmetto, FL, United States) taken ~6 h pre-trial. HR was recorded throughout, while BLa, TS, and RPE were collected pre-trial and at 10-min intervals. After the completion of the HSTs, participants exited the chamber and were instructed to rest in a seated position during which time *T*_sk_ loggers were removed and sweat patches were cleaned with deionized water and placed in collection tubes (Salivette^®^, Sarstedt, Nümbrecht, Germany) for later analysis. Capillary blood samples were then repeated as previously.

### Active Heat Acclimation

Participants were permitted a self-selected 5 min (days 1 and 8) or 15 min (days 3, 5, and 6) warm-up. Participants were provided with an individualized 5 beat min^−1^ HR zone equating to ~80% maximum HR. This intensity was chosen, based on preliminary testing and previous data (Gibson et al., 2015; Pethick et al., 2019), to elicit a *T*_c_ of ~38.5°C. Active HA sessions lasted for 45 min (days 1 and 8) or 90 min (days 3, 5, and 6). During all sessions, participants were instructed to manipulate their cycling PO, *via* the Cyclus 2, to maintain a HR within the predetermined zone. During all sessions, cycling PO was recorded and stored on the Cyclus 2 before later export and analysis. HR was recorded continuously, while BLa, TS, and RPE were collected pre-trial and at 15-min intervals.

### Passive Heat Acclimation

Passive HA sessions were performed on days 2, 4, and 7 and were structured to align with triathletes’ typical weekly running frequency when tapering for competition (Mujika, 2011). Participants were instructed to undertake their normal run training, or to run for 30 min at a moderate intensity (RPE of 13) before entering the chamber. Participants then rested in the heat for 60 min. During passive HA sessions, HR was recorded every 10 min, while *T*_sk_ was assessed *via* an insulted skin thermistor (Squirrel SQ2010, Grant Instruments Ltd., Shepreth, United Kingdom) placed at the seventh cervical vertebra (Taylor et al., 2014). Finally, TS was noted pre-trial and at 20-min intervals.

### Analytical Methods

#### Hematocrit

Hematocrit tubes were centrifuged (Haematospin 1,400, Hawksley) at 11,800 revolution min^−1^ for 5 min before being assessed *via* a tube reader (Hawksley). As samples were collected in duplicate, the mean value is presented. The coefficient of variation (CV) for duplicate samples was 1.3%.

#### Hemoglobin

A 20 μl blood samples were combined with 5 ml Drabkin’s solution with the absorbance of the resultant mixture read *via* a zeroed spectrophotometer (Cecil series 1,000, Cecil Instruments Ltd., Cambridge, United Kingdom) at 540 nm. The mean absorbance value of the duplicate samples was subsequently translated into hemoglobin concentration. The CV for duplicate samples was 2.1%.

#### Plasma Volume Changes

Changes in PV were calculated from hematocrit and hemoglobin using the equation of Dill and Costill (1974).

#### Sweat Composition

Sweat samples at all four sites were first diluted by a 1:200 ratio in deionized water before being analyzed for sodium concentration *via* flame photometry (Model 410c, Sherwood Scientific Ltd., Cambridge, United Kingdom). All individuals’ samples were analyzed in the same batch.

### Statistical Analyses

All statistical analyses were conducted using IBM SPSS Statistics 23.0 software (IBM, Armonk, NY, United States). Statistical significance was set at *p* < 0.05. Data were checked for normal distribution using the Shapiro-Wilk test, where normal distribution was not present and non-parametric test were employed. Where sphericity could not be assumed, the Greenhouse-Geisser correction was used. Differences in participants’ physical and physiological characteristics between groups were assessed *via* the Kruskal-Wallis test. To determine the likelihood of a learning effect, PO between the familiarization trial and TT1 was compared *via* paired sample *t* test. Data from the AB-CON group were used to derive the CV in PO during the performance tests without HA. From this, the smallest worthwhile change in PO was calculated (Malcata and Hopkins, 2014). PO and HR were averaged over 5 km segments during TTs, while *T*_c_, *T*_sk_, HR, and PO were averaged over 5 min segments during HSTs, active HA, and passive HA where appropriate. Changes in PO, HR, *T*_c_, *T*_*s*k_, BLa, RPE, and TS between TT1 and TT2, HST1 and HST2, active HA sessions, and between passive HA sessions were assessed *via* two-way analysis of variance or the Friedman test within groups. Changes in pre-trial USG, fluid intake, sweat rate, sweat gain, and sweat sodium concentration were evaluated by paired sample *t* test, one-way analysis of variance or Wilcoxon’s signed rank test. PV changes were assessed against a fixed zero by one-sample *t* test. Bonferroni, Mann-Whitney *U*, or Wilcoxon’s signed rank *post hoc* tests were used to identify any significant differences where appropriate. Data are presented as mean ± standard deviation (SD) where appropriate.

## Results

### Participant Characteristics

The PARA group were older than AB-CON (*p* = 0.009), but there were no significant differences in body mass, peak rate of oxygen uptake, maximum aerobic PO, or AeLT between groups (*p* ≥ 0.352; Table 1).

### Performance Tests

**Figure 2:**
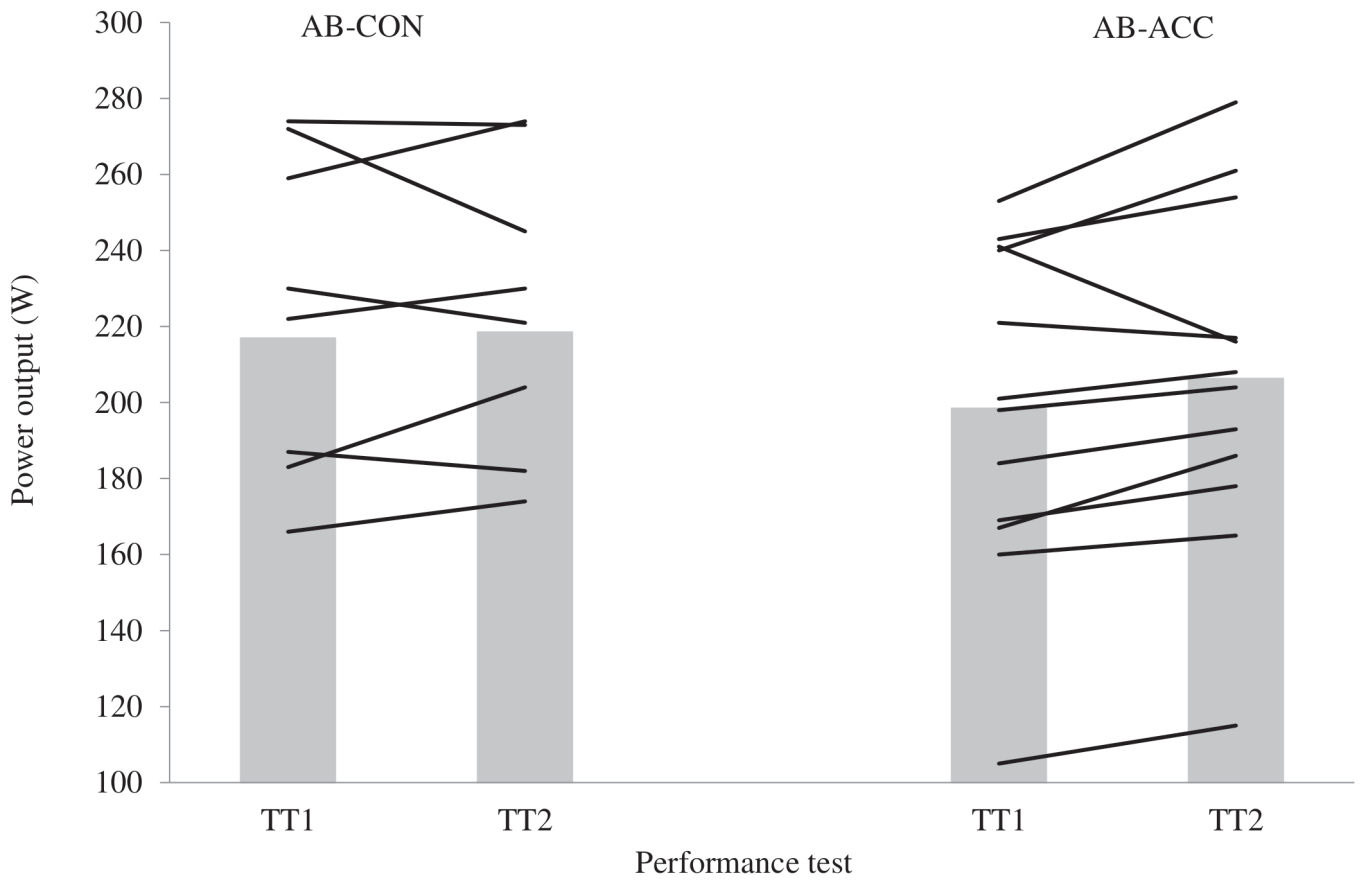
Performance trial (20 km cycling time trial in 35°C, 60% relative humidity) average power output for the control group (AB-CON) and able-bodied acclimation group (AB-ACC). Bars are group mean, and lines are individual participants.

There was no significant difference in PO between the familiarization trial and TT1 for either group (*p* ≥ 0.378). The CV in PO for AB-CON was 3.7% (TT1: 217 ± 42 W, 33.5 ± 2.4 min; TT2: 219 ± 38 W, 33.4 ± 2.1 min; Figure 2); therefore, the smallest worthwhile change in PO for TT2 was 4.3%. The average change in PO for AB was 4.5% (TT1: 199 ± 44 W, 35.3 ± 3.7 min; TT2: 207 ± 45 W, 35.0 ± 3.7 min), indicating a small meaningful improvement in average PO during the performance tests. Eight of the 12 participants in the AB-ACC group experienced an improvement in performance exceeding the smallest worthwhile change. There was no significant trial or trial by time effect for HR or BLa for either group (*p* ≥ 0.164). TS was significantly lower during TT2 for the AB-ACC group (*p* = 0.013); there was no significant change in the AB-CON group (*p* = 0.090), nor was there an effect of trial on RPE in either group (*p* ≥ 0.388). Fluid intake (TT1: 0.63 ± 0.19 L, TT2: 0.76 ± 0.25 L) and sweat loss (TT1: 1.08 ± 0.25 L, TT2: 1.13 ± 0.25 L) were not significantly different across trials for AB-CON (*p* ≥ 0.139). Fluid intake (TT1: 0.44 ± 0.22 L, TT2: 0.56 ± 0.30 L) and sweat loss (TT1: 1.08 ± 0.22, TT2: 1.21 ± 0.28) were significantly greater during TT2 for AB-ACC (*p* ≤ 0.031). There was no significant difference in pre-trial USG for either group (*p* ≥ 0.266).

### Heat Stress Tests

**Figure 3:**
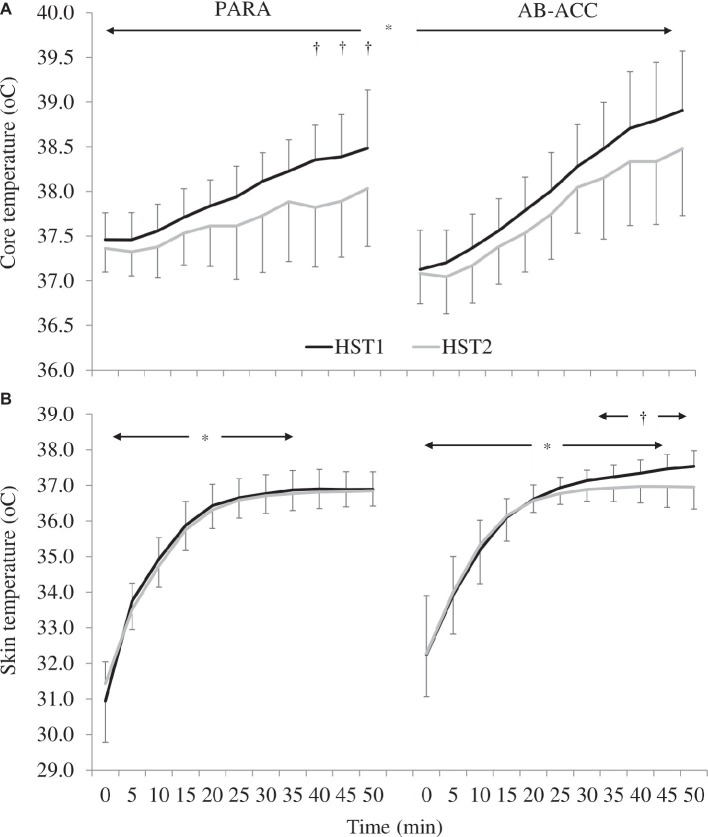
**(A)** Changes in core temperature across heat stress tests (HST) for the paratriathlon (PARA) and able-bodied (AB-ACC) group (mean ± SD). ^*^Significantly lower during HST2 and increasing over time (*p* < 0.001). ^†^Significantly lower during HST2 (*p* ≤ 0.049). **(B)** Changes in skin temperature across HST for the PARA and AB-ACC group (mean ± SD). ^*^Significant increase over time until 35 min (PARA; *p* ≤ 0.044) or 45 min (AB-ACC; *p* ≤ 0.036). ^†^Significantly lower during HST2 (*p* < 0.001).

**Figure 4:**
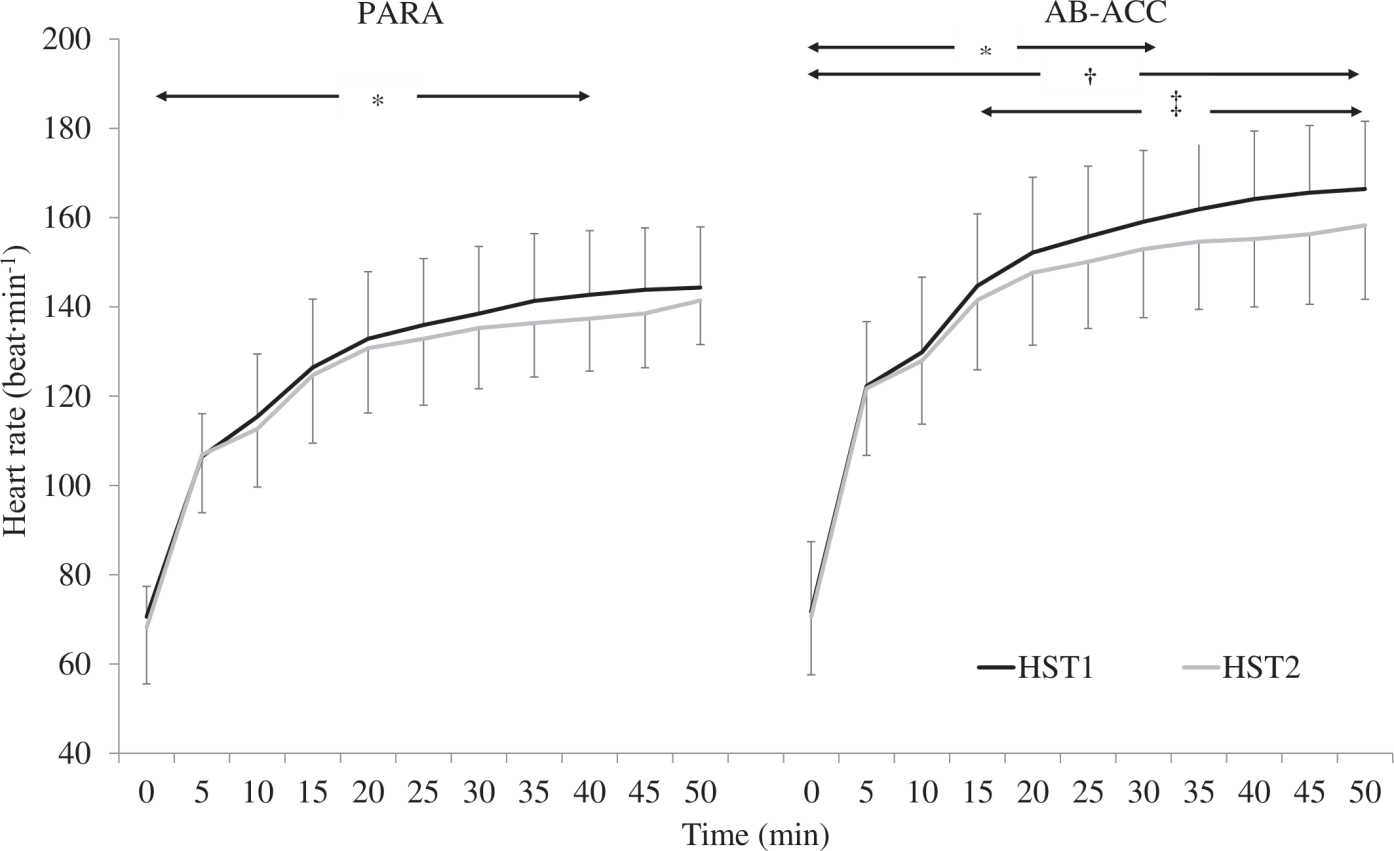
Changes in heart rate across heat stress tests (HST) for the paratriathlon (PARA) and able-bodied (AB-ACC) group (mean ± SD). ^*^Significant increase over time until 40 min (PARA; *p* ≤ 0.028) and 30 min (AB-ACC; *p* ≤ 0.031). ^†^Significantly lower during HST2 (*p* = 0.008). ^‡^Significantly lower during HST2 (*p* ≤ 0.045).

**Figure 5:**
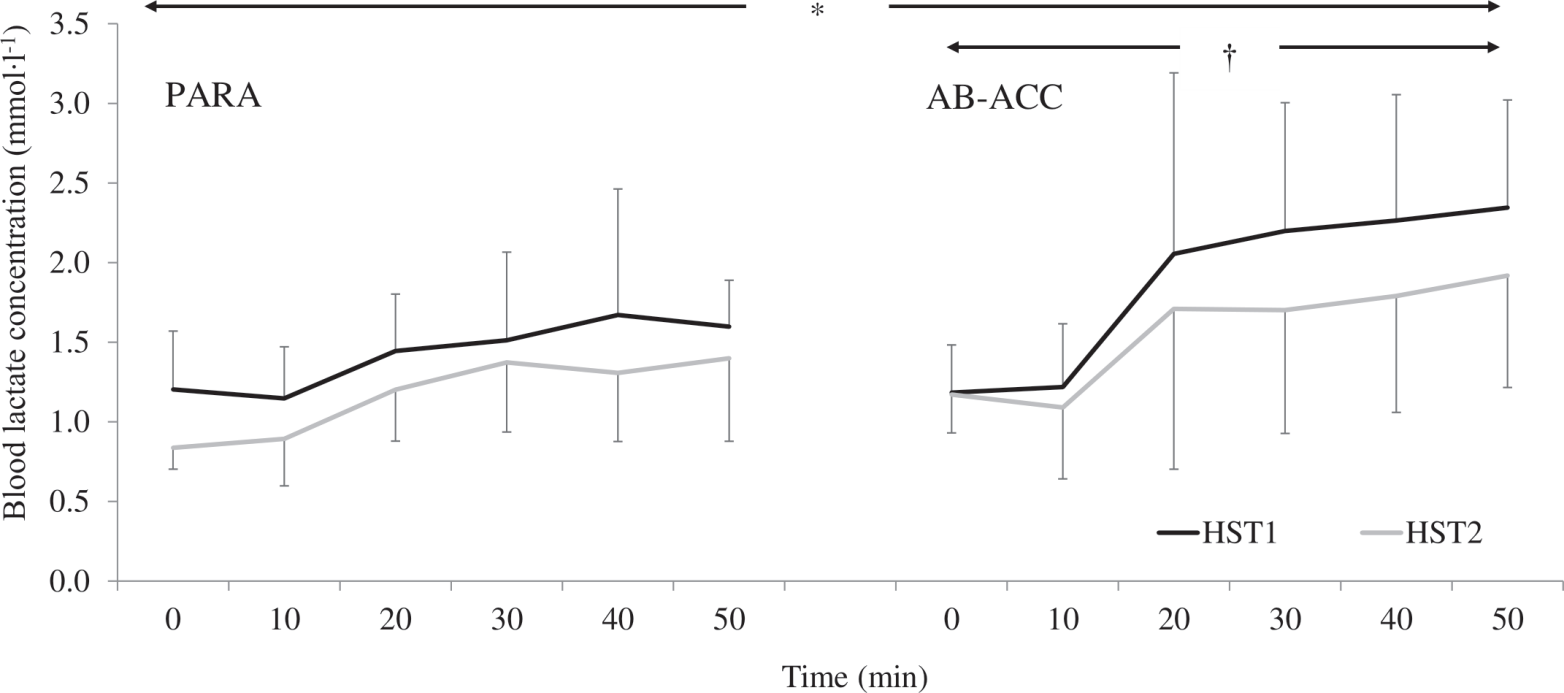
Changes in blood lactate concentration across heat stress tests (HST) for the paratriathlon (PARA) and able-bodied (AB-ACC) group (mean ± SD). ^*^Significantly lower during HST2 (*p* < 0.001). ^†^Significant increase over time (*p* ≤ 0.037).

There was a significant PV expansion for PARA (12.7 ± 10.6%; *p* = 0.019). However, there was no effect of HA on sweat rate, sweat gain, fluid intake, sweat sodium concentration, or pre-trial USG (*p* ≥ 0.066) (Table 2). For AB-ACC, there was a significant effect of trial on sweat rate, sweat gain, and fluid intake (*p* ≤ 0.045) and PV change post-HA (6.2 ± 7.7%; *p* = 0.013) with no significant change in sweat sodium concentration or pre-trial USG (*p* ≥ 0.678) (Table 2). For both groups, *T*_c_ was significantly lower in HST2 (*p* < 0.001) (Figure 3A). For PARA, there was a significant trial by time interaction, whereby *T*_c_ was lower at 40, 45, and 50 min during HST2 than HST1 (*p* ≤ 0.049). There was, however, no change in resting *T*_c_ in either group (*p* ≥ 0.367). There was no significant change in *T*_sk_ for PARA (*p* = 0.177), but *T*_sk_ was lower during HST2 for AB-ACC (*p* < 0.001); *post hoc* analyses revealed *T*_sk_ was lower from 30 min during HST2 (Figure 3B). There was no significant change in HR for PARA (*p* = 0.878), but for AB-ACC, HR was lower during HST2 (*p* = 0.008) with values lower from 15 min onward (*p* ≤ 0.045; Figure 4). For both groups, BLa (Figure 5), TS, and RPE were significantly lower during HST2 (*p* < 0.032).

**Table 2:** Pre-trial urine specific gravity (USG), sweat rate, sweat gain, fluid intake and sweat sodium (Na^+^) concentration during heat stress tests pre- (HST1) and post- (HST2) heat acclimation in paratriathlon (PARA) and able-bodied (AB-ACC) groups (mean ± SD).

	PARA		AB-ACC	
	HST1	HST2	HST1	HST2
Sweat rate (L∙h^−1^)	1.36 ± 0.73	1.49 ± 0.57	1.35 ± 0.44	1.52 ± 0.37^*^
Sweat gain (L∙h^−1^∙°C^−1^)	1.32 ± 0.53	3.33 ± 2.37	0.74 ± 0.28	1.19 ± 0.90^*^
Fluid intake (L∙h^−1^)	0.88 ± 0.40	0.72 ± 0.16	0.82 ± 0.43	1.04 ± 0.55^*^
Average sweat Na^+^ (mmol∙L^−1^)	37.1 ± 7.1	40.7 ± 12.5	49.2 ± 16.1	48.1 ± 20.6
Pre-trial USG	1.019 ± 0.004	1.022 ± 0.007	1.014 ± 0.008	1.015 ± 0.008

* *Significantly different to HST1 (*p* ≤ 0.045)*.

### Active Heat Acclimation

**Figure 6:**
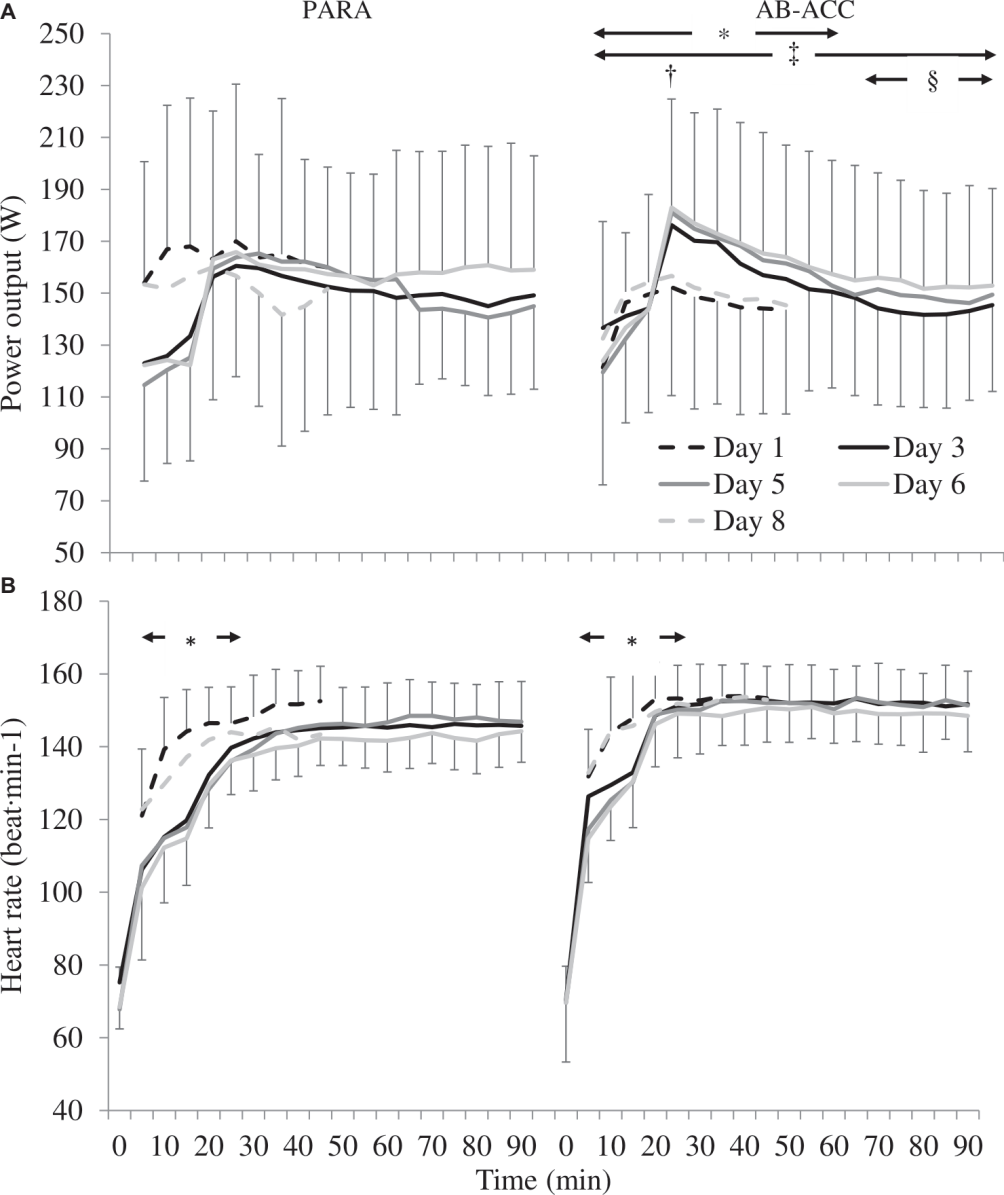
**(A)** Power output changes between and within active heat acclimation sessions for paratriathlon (PARA) and able-bodied (AB-ACC) groups (mean ± SD). ^*^Significant difference between days 1 and 8 (*p* = 0.048). ^†^Significantly greater at 20 min than 5 min during days 1 and 8 (*p* = 0.010). ^‡^Significantly greater than day 3 (*p* ≤ 0.021). ^§^Significantly greater than day 6 (*p* ≤ 0.044). **(B)** Heart rate changes between and within active heat acclimation sessions for PARA and AB-ACC groups (mean ± SD). ^*^Significant increase up to 20 min (*p* ≤ 0.040).

There was no significant difference in PO between days 1 and 8 (45 min) for PARA (*p* = 0.522), but for AB-ACC, it was higher on day 8 (*p* = 0.048). Comparing days 3, 5, and 6 (90 min), there was no meaningful difference in PO between trials for PARA (*p* = 0.483). However, for AB-ACC, *post hoc* analyses revealed PO was higher on days 5 and 6 than day 3 (*p* ≤ 0.021) and was greater on day 6 than days 3 and 5 from 65 min onward (*p* ≤ 0.044) (Figure 6). There was no significant difference in HR, BLa, sweat rate, fluid intake, or RPE between active HA sessions for either group (*p* ≥ 0.068). For AB-ACC, TS was lower on day 5 than days 3 and 6 (*p* ≤ 0.026). There was no change in TS over time for PARA (*p* ≥ 0.450; Table 3).

**Table 3:** Blood lactate concentration (BLa), thermal sensation (TS), rating of perceived exertion (RPE), sweat rate, and fluid intake changes between active heat acclimation sessions for paratriathlon (PARA) and able-bodied (AB-ACC) groups (daily mean ± SD).

	Day 1	Day 3	Day 5	Day 6	Day 8
**PARA**					
BLa (mmol∙L^−1^)	1.75 ± 0.87	1.54 ± 0.51	1.60 ± 0.69	1.49 ± 0.56	1.63 ± 0.89
TS (AU)	6 ± 1	5 ± 2	5 ± 2	5 ± 2	6 ± 1
RPE (AU)	13 ± 1	12 ± 2	12 ± 1	12 ± 1	13 ± 1
Sweat rate (L∙h^−1^)	1.40 ± 0.50	1.29 ± 0.52	1.19 ± 0.47	1.33 ± 0.52	1.57 ± 0.45
Fluid intake (L∙h^−1^)	1.38 ± 0.90	1.28 ± 0.62	1.30 ± 0.50	1.29 ± 0.56	1.65 ± 0.51
**AB-ACC**
BLa (mmol∙L^−1^)	1.28 ± 0.27	1.44 ± 0.52	1.47 ± 0.62	1.37 ± 0.52	1.78 ± 0.91
TS (AU)	7 ± 1	6 ± 2	6 ± 2^*^	6 ± 2	6 ± 2
RPE (AU)	14 ± 1	13 ± 2	13 ± 2	13 ± 2	13 ± 2
Sweat rate (L∙h^−1^)	1.31 ± 0.45	1.22 ± 0.40	1.32 ± 0.36	1.35 ± 0.39	1.27 ± 0.81
Fluid intake (L∙h^−1^)	1.46 ± 0.77	1.22 ± 0.54	1.31 ± 0.57	1.45 ± 0.64	1.54 ± 0.48

* *TS significantly lower on day 5 than days 3 and 6 in AB-ACC (*p* ≤ 0.026)*.

### Passive Heat Acclimation

There was no change in HR or *T*_sk_ across trials for either group (*p* ≥ 0.224; Table 4). TS was greater at 60 min than 0 and 20 min (*p* ≤ 0.032) and was greater on day 2 than days 4 and 7 in PARA (*p* ≤ 0.003). For AB-ACC, HR was greater at 0 min than all other time points (*p* ≤ 0.038), and *T*_sk_ was lower at 0 min than 10 min (*p* = 0.032).

**Table 4:** Heart rate (HR), skin temperature (*T*_sk_), and thermal sensation (TS) between passive heat acclimation sessions for paratriathlon (PARA) and able-bodied (AB-ACC) groups (daily mean ± SD).

	PARA			AB-ACC		
	Day 2	Day 4	Day 7	Day 2	Day 4	Day 7
HR (beat∙min^−1^)	74 ± 8	75 ± 8	75 ± 9	78 ± 13	77 ± 11	78 ± 14
*T*_sk_ (°C)	35.27 ± 1.07	35.21 ± 0.88	35.13 ± 0.66	34.76 ± 1.22	35.00 ± 0.87	35.08 ± 0.71
TS (AU)	4 ± 1	3 ± 1	3 ± 1	4 ± 2	4 ± 2	4 ± 2

*TS greater on day 2 than days 4 and 7 in PARA (*p* ≤ 0.003)*.

## Discussion

This is the first study to investigate the efficacy of HA in a multi-impairment, Paralympic endurance sport. Utilizing a novel, mixed active and passive HA protocol controlling relative intensity through HR, paratriathletes can display positive thermoregulatory adaptations. These responses were a reduction in exercising *T*_c_, BLa, RPE, and TS with concomitant PV expansion. Furthermore, additional adaptations were noted including *T*_sk_, HR, and sudomotor changes during the submaximal HST in a physiologically matched AB group. Finally, there was a small direct performance benefit from the employed HA protocol as AB-ACC athletes improved their PO during a 20 km cycling TT in the heat (4.5%) to an extent greater than the variation noted in a non-acclimated AB-CON group (3.7%).

Medium-term HA (8–14-day heat exposure) has been commonly studied in the literature with typical adaptations recently documented in the meta-analysis of Tyler et al. (2016). This approach has been shown to result in a decrease in exercising: *T*_c_ (0.17°C); HR (15 beat min^−1^), *T*_sk_ (0.73°C), BLa (~0.9 mmol∙l^−1^), TS, and RPE (~10%); with increased whole-body sweat rates (30.0%) and PV expansion (4.3%; Tyler et al., 2016). Thus, this study presents thermoregulatory adaptations for both groups that are within the commonly reported range for similar duration HA, while the greater *T*_c_ (~0.28°C) and PV (6.2–12.7%) adaptations here may be a result of the study design ensuring a consistent thermal impulse and relative exercise intensity. Finally, medium-term HA also results in a median 4% improvement in TT performance (Tyler et al., 2016), similar to the 4.5% improvement noted in the AB-ACC group. A noteworthy finding of the current study is the greater magnitude of PV adaptation for PARA relative to AB-ACC. The exact reason behind this response is unknown as this suggests a greater thermal impulse in PARA, which could not be confirmed due to a lack of *T*_c_ measurement during HA. Furthermore, HA typically results in a 0.18°C reduction in resting *T*_c_ (Tyler et al., 2016); but no reduction was seen presently. This is likely due to a lack of rigid control pre-trial to accurately detect changes in truly resting *T*_c_.

As noted earlier, the literature to date concerning HA in Paralympic athletes has been confined to one study in target shooters with a spinal cord injury (Castle et al., 2013), despite acknowledgments of the need for greater evidence (Price, 2015; Casadio et al., 2017). The authors stated that the five participants displayed partial HA through a decrease in resting and exercising aural temperature, end-exercise HR, TS, and RPE. These responses were proposed to be mediated by a 1.5 ± 0.6% PV expansion. There were, however, no notable sweat responses, which were credited to the nature of athletes’ autonomic impairments. In the present study, very similar HA adaptations were noted; however, the extent of PV was greater and more varied. This is most likely attributed to the disparate athlete impairments, the HA protocol presenting a greater thermal stimulus, and the incongruent athletic backgrounds. Thus, the present data provide support to the notion that Paralympic athletes are capable of partial HA although not to the same extent as AB athletes undergoing the same protocol. While the current study contained a range of physical impairments in the PARA group, the small sample size did not permit meaningful comparisons between impairments. As such, we cannot differentiate impairment-specific adaptations from individual variability. Nonetheless, analysis of individual data suggests those with presumably greatest thermoregulatory impairment (an athlete with spinal cord injury) can display beneficial adaptions to HA with improvements in *T*_c_, HR, BLa, and PV noted. As such, there is currently no reason why all Paralympic endurance athletes should not undergo HA before competition in the heat.

A unique feature of this work was that differences in HA responses between AB and Paralympic athletes were present. It is worth noting, however, that the small and heterogeneous sample size in the PARA group may have prevented some statistically significant findings. For example, improvements in HR and whole-body sweat rates during exercise were noted; however, these did not reach the predefined threshold of significance. Alternatively, the nature of physical impairments in the PARA group may have restricted any notable adaptation. For instance, the reduced body surface area for heat dissipation of those with an amputation may be the limiting factor in any *T*_sk_ change, rather than PV and capacity for skin blood flow. Further, limited sweating responses as a result of reduced body surface area, skin grafts, or disrupted afferent input likely reduce the maximum achievable sweating capacity, thus creating a “ceiling” effect. Nonetheless, improvements in exercising *T*_c_, BLa, and perceptual measures demonstrated an enhanced thermoregulatory capacity, which was likely mediated by a significant PV expansion post-HA. While not directly assessed, it may be assumed that this would result in a direct improvement in endurance performance in hot environments as athletes display a diminished heat storage and reliance upon carbohydrate metabolism. Of note, while the PARA was older, this was not considered a major concern since the previous work has shown age that does not affect thermoregulatory or performance changes post-HA (Tyler et al., 2016).

The use of HR to regulate HA training intensity was based on the recommendations of Périard et al. (2015) to provide a constant cardiovascular, and presumed thermoregulatory, stimulus for continued adaptation. This is supported by previous work showing a constant HR across isothermic HA (Magalhães et al., 2010; Garrett et al., 2012; Zurawlew et al., 2015; Pethick et al., 2019). While isothermic protocols have been commonly utilized in the literature to provide a constant thermal stimulus (Tyler et al., 2016), the utility of such approaches has been questioned in the applied sport setting. Therefore, the use of HR provides a feasible means for regulating HA intensity for elite athletes. Participants in the present study were able to maintain their individualized HR zone within and between HA sessions, and as the target HR was relative to their maximum HR, the relative intensity of exercise was the same between groups. Furthermore, due to a lack of change in BLa, RPE, and TS between active HA, participants maintained a constant relative intensity each day. Moreover, the AB-ACC group was able to produce a greater external workload in later HA sessions suggesting that the group displayed thermoregulatory adaptations that permitted a higher workload for a given relative intensity. This is akin to isothermic HA, whereby greater workloads are produced for a set *T*_c_ (Magalhães et al., 2010; Garrett et al., 2012; Pethick et al., 2019). However, no significant change in PO was noted for the PARA group. This may be indicative of the greater individual variation in this group with regard to their physical impairments, exercising workload, and capacity for adaptation.

The current protocol was chosen to provide an optimal yet time efficient HA stimulus when considering elite athletes’ pre-competition training schedules (Guy et al., 2014; Casadio et al., 2017). A combined HA approach may present a pragmatic solution to overcome the demands of pre-competition schedules while potentiating a stimulus for heat adaptation (Ruddock et al., 2016), although this is severely understudied. However, Ruddock et al. (2016) utilized this study design in their case study of a soccer referee. The authors showed that the participant displayed increases in whole-body sweat rate, PV, and repeated sprint performance while decreasing exercising tympanic temperature and HR. In the present study, passive heat exposure was in the form of post-exercise rest in a climatic chamber. This permitted athletes the capability to maintain running session density and intensity while continuing daily heat exposure as athletes entered the heat with a presumed prior elevated *T*_c_, *T*_sk_, and sweat rate. While *T*_sk_ did not reach the levels reported by Zurawlew et al. (2015) (equilibrating at 40°C water temperature) during passive heating, it was greater than typical resting temperatures, and the reported TS further indicated some thermal strain. Indeed, as beneficial adaptations were noted in the present study for both AB-ACC and PARA, this adds support for the use of mixed active and passive HA for elite athletes, pre-competition. However, it is unknown if a solely active or passive approach would have induced disparate adaptations.

The timings of this study meant that the PARA group undertook their HA sessions in preparation for a competitive race in the heat. Therefore, it was neither feasible nor appropriate to include a direct performance test for this group. However, this was included in the AB-ACC and AB-CON groups. The AB-ACC group displayed a small improvement in average PO, thus providing evidence for a direct performance benefit of the HA protocol. Furthermore, the AB-ACC group displayed physiological responses during TT2 indicative of an enhanced thermoregulatory capacity. Specifically, a greater sweat rate and fluid intake was noted, while participants produced a greater PO for a similar HR and lower TS, whereas no significant changes were noted for the AB-CON group. It can therefore be assumed that these thermoregulatory adaptations, among others, permitted the improvement in performance.

Here, the lack of a control group engaging in exercise in thermoneutral conditions prevented the ability to state that changes in thermoregulatory variables were due solely to HA. Nonetheless, due to the relatively low HA intensity and the high cardiorespiratory fitness levels of both PARA and AB-ACC groups, it is unlikely any non-HA-specific training adaptation occurred (Lorenzo et al., 2010). However, there is the potential that HA resulted in a positive phenotypic adaptation in athletes’ AeLT and/or maximum rate of oxygen uptake. This has been shown by previous research (Lorenzo et al., 2010; Corbett et al., 2014; Tyler et al., 2016), although not all (Chalmers et al., 2014; Neal et al., 2016), and may have reduced the metabolic heat production during HST2. As post-HA graded exercise tests were not conducted and gas exchange variables were not measured during HSTs, this could not be confirmed. Furthermore, it is noteworthy that *T*_c_ was not recorded across exercise HA. Although both groups maintained a constant relative intensity, assuming a constant thermal stimulus, this was not confirmed. Lastly, the current study did not control for menstrual cycle phase in female participants, which may have influenced *T*_c_ (Casadio et al., 2017).

## Conclusions

A mixed active and passive HA protocol, controlling exercise intensity by HR, induced positive thermoregulatory adaptations in paratriathletes and AB triathletes. Both groups displayed reductions in *T*_c_, BLa, RPE, and TS during a submaximal HST with significant PV expansion. Furthermore, the AB-ACC group presented additional adaptations including reduced HR and *T*_sk_ with an elevated sweat rate. This is the first evidence of differences in thermoregulatory variable changes to the same protocol between Paralympic and AB athletes matched for physiological variables. The HR-controlled exercise HA resulted in a constant relative intensity between HA sessions with the AB-ACC group capable of producing a greater PO for a set intensity over the study period. Finally, there was evidence of a direct performance benefit as the AB group improved their PO during a 20 km TT in the heat to a greater extent than the natural variation shown in a non-acclimated, matched cohort.

## Data Availability Statement

The datasets generated for this study are available on request to the corresponding author.

## Ethics Statement

The studies involving human participants were reviewed and approved by Human Participants Sub-Committee Loughborough University. The patients/participants provided their written informed consent to participate in this study.

## Author Contributions

BS and VG-T designed the study. BS collected and analyzed the study data. BS and KT undertook the statistical analyses. BS wrote the manuscript, which was edited by KT and VG-T.

### Conflict of Interest

The authors declare that the research was conducted in the absence of any commercial or financial relationships that could be construed as a potential conflict of interest.

## Abbreviations

AB: Able-bodied
AeLT: Aerobic lactate threshold
BLa: Blood lactate concentration
CV: Coefficient of variation
HA: Heat acclimation
HR: Heart rate
PO: Cycling power output
RPE: Rating of perceived exertion
PV: Plasma volume
SD: Standard deviation
*T*_c_: Core temperature
TS: Thermal sensation
*T*_sk_: Skin temperature
USG: Urine specific gravity.
